# A Framework for Sustainable Implementation of E-Medicine in Transitioning Countries

**DOI:** 10.1155/2013/615617

**Published:** 2013-12-26

**Authors:** Stephen Robert Isabalija, Victor Mbarika, Geoffrey Mayoka Kituyi

**Affiliations:** ^1^Department of Business Administration, Makerere University Business School, 1337 Kampala, Uganda; ^2^International Center for Information Technology and Development, Southern University and A and M College, P.O. Box 9723, Baton Rouge, LA 70813-9723, USA; ^3^Department of Business Computing, Makerere University Business School, 1337 Kampala, Uganda

## Abstract

Organizations in developed countries such as the United States of America and Canada face difficulties and challenges in technology transfer from one organization to another; the complexity of problems easily compounds when such transfers are attempted from developed to developing countries due to differing socioeconomic and cultural environments. There is a gap in the formation of research and education programs to address technology transfer issues that go beyond just transferring the technologies to sustaining such transfers for longer periods. This study examined telemedicine transfer challenges in three Sub-Sahara African countries and developed a framework for sustainable implementation of e-medicine. Both quantitative and qualitative research methods were used. The study findings indicate that e-medicine sustainability in Sub-Saharan Africa is affected by institutional factors such as institutional environment and knowledge management practices; technical factors such as the technological environment and technology transfer project environment; social environmental factors such as social environment and donor involvement. These factors were used to model the proposed framework.

## 1. Introduction

Healthcare is unarguably one of the most fundamental needs for Sub-Saharan Africa (SSA), considering the region's multiple medical problems. The health statistics of SSA are deplorable. The academic and practitioner literature report many medical problems of SSA. Yet SSA is the most vulnerable to disease, given the prevalent social, economic, and environmental factors. For instance, the World Health Organization (WHO) reports that by the end of year 2009, over 32.9 million people worldwide were living with HIV/AIDS. Out of these, 22 million (approximately 68%) live in SSA [1]. By the end of year 2009, the percentages of people living with HIV/AIDS in Botswana, Central African Republic, and Swaziland were still the highest in the world [1, 2].

Further to the above, out of the estimated 9.7 million number of children under the age of five who die every year due to lack of access to medical facilities worldwide, 41% live in SSA. Research shows that malaria is responsible for as many as half the deaths of African children under the age of five. This disease kills more than one million children (2,800 per day) each year in Africa alone. In regions of intense transmission, 40% of toddlers may die of acute malaria. In most malaria cases, however, there is a good chance of survival if timely and appropriate medical attention is provided. Other diseases that plague the continent and lead to the loss of millions of lives every year in Africa include dysentery, cholera, typhoid, yellow fever, and diarrhea [1].

One notable challenge in SSA is the shortage of medical personnel and facilities. Many developing countries have an acute shortage of doctors, particularly specialists. SSA has, on average, fewer than 10 doctors per 100,000 people, and 14 countries do not have a single radiologist [3, 4]. The specialists and services that are available are concentrated in large urban cities.

In the healthcare sector, rising costs and new types of health problems result in increasing pressure on the healthcare system and stimulate new approaches for improving access and reducing the cost of healthcare [5, 6]. Telemedicine initiatives represent potential solutions to improve healthcare accessibility and quality. Modern day healthcare is offering more and more treatment alternatives on the Internet, called e-medicine also sometimes generally referred to as telemedicine [7].

E-medicine is one of the most powerful initiatives for enabling access to health services in rural areas which are in most cases hard-to-reach. E-medicine is the use electronic means to transfer medical data from one place to another. At advanced levels, e-medicine may involve conducting clinical practices using telecommunication facilities such as teleconferencing. Simple applications of e-medicine may be manifested in medical record keeping, data processing, and information sharing. At a lower level e-medicine may also involve teleconsultation, whereby health workers can offer consultancy services to peers and/or patients [1]. In this study, our emphasis was placed on the use of health information systems, teleconferencing facilities, and medical data processing application in the hospitals. Studies by [8, 9] and [3] show that these are the most commonly used e-medicine systems in SSA.

The high penetration of mobile devices and networks globally implies that mobile technologies can be used very effectively in the field of healthcare in order to compensate for the scarcity of resources, particularly in developing countries [10]. In general, e-medicine has advantages where there is relatively inadequate or nonexistent access to healthcare resources, uneven geographical distribution of expertise, and continuing increases in the cost of healthcare services. In these circumstances, e-medicine improves access to healthcare, reducing the cost. Also, by improving communication between health centers (peripheral) and secondary or tertiary hospitals, e-medicine has been shown to speed up the referral process, reduce unnecessary referrals, and improve quality of care [3, 9, 11]. In some cases, e-medicine may be cheaper than the conventional practice [9].

The advent of e-medicine has presented numerous opportunities for countries without adequate human resources to benefit from global manpower that resides in the developed world. However, many telemedicine projects initiated in Sub-Saharan Africa have always failed without tangible benefits. One of the causes of failure is because there are inappropriate telemedicine implementation frameworks [9]. Scholars [12] argue that there should be telemedicine implementation and sustainability frameworks tailored to the local needs of countries in SSA. The purpose of the study therefore was to develop a framework which can facilitate the development, implementation, and sustainability of e-medicine in SSA employing a mixed research approach.

## 2. Materials

This section presents a brief review relevant literature that was consulted to enrich and ground the study on theory. The section presents the contextual analysis of Sub-Saharan Africa and examines the sustainability network theory, the technological environment, and the social environment factors that influence the sustainability of e-medicine outcomes in transitioning countries, especially Sub-Saharan Africa.

### 2.1. Overview of Sub-Saharan Africa

Sub-Saharan Africa is the part of the African continent that stretches out from Senegal, Niger, Mali, Chad, Djibouti, and Ethiopia, coming southwards to South Africa. It is sometimes referred to as the Black Africa because most of its inhabitants are black. The majority of people in SSA form part of the world's poorest people, whereby over 60% live below the poverty line of $1 per day. In addition, the region is characterized by high birth and death rates with low life expectancy (between 45 and 55 years) in different countries. For example, the United Nations study puts birth rates from years 2005 to 2010 for SSA at 5.1 per woman [13]. Further, [13] estimates that the population for SSA region will shoot from 0.86 billion people in year 2010 to 1.96 billion people in year 2050. This means that there is greater need for efficiency in healthcare service delivery, which requires e-medicine. Moreover, studies on the uptake of e-medicine and telemedicine in this region have revealed appalling findings as most projects do not live even for a year [3, 8, 9].

### 2.2. Sustainability Network Theory

According to [14], sustainability networks concern properties that arise in systems of many objects linked together and displaying both static and dynamic complexity. From a static perspective, networks are characterized by a number of key concepts such as connectivity (nodes, links, and flows), criticality, loops and cycles, dynamics, modularity, trees, and hierarchies. But it is the dynamics of industrial systems that are particularly challenging, and it is here that the need for sustainability network theory (SNT) [14] becomes apparent, because many of the behaviors of such systems arise not from the substantive factors that are the usual focus of analysis but from their underlying networks structure and dynamics. Thus, tightly coupled networks are more resistant to change than loosely coupled networks. This is a characteristic of complex systems that explains why changes to pollution control equipment regulations are more easily accomplished than changes to product design or manufacturing process regulations. In the former case, the technology is only loosely coupled to underlying product and manufacturing networks and thus can be changed with only minimal implications for other aspects of the product and manufacturing networks.

### 2.3. The Technological Environment

The technological environment largely involves the current state of ICT infrastructure in a country [15]. This factor impacts transfer of e-medicine to developing countries in terms of the basic ICT infrastructures, such as levels of basic telephone penetration (teledensity—the number of land telephone lines per capita). A country needs a solid ICT infrastructure for telemedicine to be possible. Due to various socioeconomic and political problems, SSA has the lowest levels of ICT-related infrastructures in the world [12]. SSA countries share a common set of problems regarding ICT, among which are a huge gap between supply and demand, a strong distribution imbalance favoring urban over rural areas, poor quality of service, long waiting times for new services, and peak traffic demands that exceed network capacity [12]. These problems result in extremely low levels of basic telephone penetration, which remain the base framework for both voice and data communications.

Overall, current models of ICT transfer within developed countries assume an existing ICT infrastructure on which applications such as telemedicine can be built. This is far from reality in SSA continues and remains a major bottleneck to e-medicine transfer within the region. Although some telemedicine projects have succeeded in SSA, these projects have been less sophisticated such as in teleradiology that does not require real-time transmission of X-ray images. The region is therefore limited to store-and-forward telemedicine practices as opposed to more sophisticated and needed practices such as telesurgery, which requires real-time transmission. Yet less sophistication or complexity is a viable approach to the current problem in the short run; however, for long-term solutions, SSA countries must expend efforts to improve on their underlying technology infrastructure so that they can enjoy more state-of-the-art telemedicine technologies.

### 2.4. Social Environment

Social environment encompasses the factors that influence the uptake of technologies emanating from the community where the technology is implemented. Social environment model is a traditional ICT transfer model that theorizes that, in general, three general classes of precursors are required for successful transfer: the existence of the specific ICTs that need to be transferred; a basic ICT infrastructure to support the new technology; and appropriate implementation of the transfer project [16]. As in any project, it is imperative that the underlying processes be optimized before initiating ICTs. For regions such as SSA, this means revamping current healthcare practices and system from their present glut of bureaucracy, corruption, and social stigmas to a coordinated flow of materials and information. For example, in anticipation of violence, ostracism, and even murder, many people in SSA fear to reveal their ailments. In cases where conditions are diagnosed, rampant bureaucracy and corruption result in gross delays in delivery of solutions.

While it is generally taken for granted that traditional healthcare is in a functional state in most developed countries (relative to developing nations), the effectiveness of the system in developing countries as those in SSA is an important consideration in the potential effectiveness of telemedicine. In a study of developed countries, the prior state and effectiveness of the national healthcare industry—encompassing, for example, hospitals and clinics, health professionals, effectiveness, and efficiency of health administrations—would never be tested for, but this must be explicit in a model of the effects of telemedicine in SSA. ICT transfer models created in developed countries assume that the transfer domain is already healthy and just needs to be wired (or unwired) with ICTs to make it even better. However, when the base domain is in poor condition, as in many developing countries, successful transfer is jeopardized by this fact alone. Thus, we contend that telemedicine projects supported by a more robust national healthcare system are likely to produce more favorable outcomes compared to telemedicine projects that lack support of a robust healthcare system.

### 2.5. Sustainability of E-Medicine Outcomes in Transitioning Countries

The challenges faced by developing nations are complex, cutting across all sectors of society, and they can be addressed effectively only by implementation of transformative and sustainable change. Prerequisites for such change include a long-term commitment to system reform and resource development. Partnerships focused on capacity building can serve as important resources for the needed change [17]. Sustainability is the capacity of programs to continuously respond to community issues and maintenance of focus on set goals. Achieving sustainability has become a central issue of program implementation. The challenges of how we can become sustainable continue to simmer over basic issues such as what it even means to be sustainable and what new knowledge is required to become sustainable [18]. We look at sustainability in terms of how programs contract and others expand. Whereas some programs maintain original program activities, some become aligned with other organizations and established institutions, and still others maintain their independence. The key element of sustainability is providing continued benefits, regardless of particular activities delivered or the format (institutionalization versus independence) in which they are delivered. Thus, it is more important to sustain benefits to communities than to sustain program activities per se.

The emerging field of sustainability science provides a fresh perspective on learning because of its focus on several major learning challenges in policy and sustainable development [19]. Scholars in this community generally agree that learning is a critical hinge for sustainability [20], but how we get there is another problem. So far there has been no systematic treatment of learning for sustainability. Despite some attempts to outline a comprehensive research program, for example, [20], the development of strategies to promote learning for sustainability remains an elusive goal. Many scholars recognize the need for institutions that promote learning in the face of complex and uncertain problems.

A growing literature on “collaborative policy,” for example, argues that networks spanning otherwise fragmented groups of stakeholders promote an effective exchange of information and the learning of common worldviews. However, there is sparse evidence that those collaborative institutions and the social networks they produce actually promote learning and improve outcomes. This underscores a central problem with the literature on institutional design to promote sustainability. The process of learning is often treated as a black box, and the design of strategies to promote learning is thus based primarily on anecdotal evidence rather than on lessons from theoretically grounded and empirically based models. A better understanding of how and why agents learn, including a detailed map of the parameters that influence this process, is a prerequisite for thinking about the types of institutions that are needed to promote learning. Understanding which types of actors are most likely to engage in constructive discussion, for example, will inform decisions regarding whom to invite to participate in a shared learning space, such as a scientific assessment process or a collaborative planning effort. These decisions must be based on a stronger theoretical and empirical understanding of how and why learning occurs. In order to accomplish this, however, we need an integrative framework that resolves the confusions and contradictions that often surround the study of learning.

One idea is that the most basic framework for understanding sustainability does not rely on understanding the interrelationship between its principal values (ecosystem health, social justice, and human needs); rather, the most basic framework for understanding sustainability may be the interrelationship between its technical and philosophical dimensions. These dimensions were dubbed by [21] as the substantive and no substantive aspects of sustainability, respectively. By this assessment, the technical dimension seems valuable for its ability to define problems precisely and to be usefully applied to many specific cases that differ greatly in circumstance (e.g., achieving a sustainable harvest of some particular population or achieving sustainable water use in some local community). This value is clearly demonstrated by the framework that supports sustainability science [22].

For sustainability of e-medicine outcomes, we note that standard knowledge hierarchy of data, information, and knowledge—in which data or simple facts become information when they are interpreted and become meaningful, and information becomes knowledge when it is put into a larger context—has been challenged by the construction of a reversed knowledge hierarchy that argues that data and information emerge only after knowledge is already available. But when the knowledge creation process is seen as a temporal sequence, sustainability is questioned [23]. These views can be combined without contradiction that previous knowledge is required to organize methodologically the production of new data or information and to interpret data and information for sustainability.

We examine seven major elements of sustainability: leadership competence, effective collaboration, understanding the community, demonstrating program results, strategic funding, staff involvement and integration, and program responsivity. These elements are mainly within the control of program leaders and stakeholders, but a program may have limited life because of factors outside the control of the program, such as state or local budget shortfalls or the emergence of other programs and organizations [24].

## 3. Methods

### 3.1. A Mixed Research Approach

A combination of methods and the sequence of the methods chosen in any particular study are critical decisions that were informed not just by the research question but also by the researchers' epistemological commitment and ontological views. Based on existing different epistemological assumptions, information system and social sciences research utilizes both quantitative and qualitative methods [25, 26]. Quantitative research methods were preferred because the research findings will apply to more than one population, thereby increasing the possibility of generalizing the research findings.

For purposes of this study, the research questions required systematically a variety of methods and techniques. Multiple methods were used in obtaining the findings [27, 28] in this case, the sustainability of e-medicine in SSA which links well with the guiding theoretical framework of this research. The focus was on studying the impacts of key factors within specific policy implementation, infrastructural, and knowledge management, with an overall goal of constructing a model that captures the synergistic relationships among key domain factors influencing sustainable e-medicine outcomes.

### 3.2. Methods Used to Improve Trustworthiness Criteria in Qualitative Methods

Research within a natural inquiry approach is guided by specific criteria to establish its validity (traditional criteria) or trustworthiness [29, 30]. The study examined four levels in trustworthiness: credibility (internal validity), transferability (external validity), conformability (objectivity), and dependability (reliability). This is summarized in Table 1.

In establishing the true value of findings, credibility was established through using two sources and cross-checking data. Transferability was considered by making certain claims that were limited by sample characteristics and providing a thick description of concepts and categories. Conformability, which requires that findings would emerge again if the study were conducted with similar situations, was maintained with careful management of data. All interviews were transcribed verbatim and clearly labeled as per respondent. To warrant that the findings reflect the interpretation of the participants, dependability was ensured through clear record of the study which was recorded and stored supported with snowballing.

### 3.3. Sample Size

A sample of 416 was chosen purposively from Uganda (sample = 135), Ethiopia (sample = 131), and Nigeria (sampler = 150) to participate in this study. These included hospital administrative and ICT staff.

In Uganda, the sample distribution included 25 doctors (10 from Nsambya hospital and 10 from Mulago hospital), 58 nurses (32 from Nsambya hospital and 26 from Mulago hospital), 38 hospital administrators (20 from Nsambya hospital and 18 from Mulago hospital), and 27 information technology/information systems employees (15 from Nsambya hospital and 12 from Mulago hospital).

In Nigeria, 70 respondents came from Pan-African Telemedicine, University of Ibadan teaching hospital, whereas 80 came from Lagos hospital. In Pan-African Telemedicine, University of Ibadan teaching hospital, the sample was distributed as follows: 18 medical doctors, 25 nurses, 12 hospital administrators, and 15 information technology/information systems employees. In Lagos hospital, the sample was distributed as follows: 20 medical doctors, 30 nurses, 15 hospital administrators, and 15 information technology/information systems employees, while in Ethiopia, the sample 130 came from Bethel teaching hospital and was distributed as follows: 15 medical doctors, 50 nurses, 45 hospital administrators and 26 information technology/information systems employees.

The above sample was selected using purposive sampling method, which is nonprobability sampling method. There are sound theoretical reasons why most qualitative research uses non-probability sampling techniques and good practical reasons why qualitative researchers deal with small numbers of instances to be researched.

The sample size is unlikely to be known with precision or certainty at the start of the study. Second, the sample size will generally be very small. Both points can be unnerving. They go against the grain as far as conventional survey approaches are concerned and open up the prospect of accusations of sloppy and biased research design. The researcher is quite explicit about the use of non-probability sampling [31, 32]. Another point is that phenomenology is well suited to purposeful sampling. This type of sampling permits the selection of interviewees whose qualities or experiences permit an understanding of the phenomenon in question and are therefore valuable. This is the strength of snowballing.

It is purely for this reason that the researcher decided to interview 10 participants with special qualifications for the study per country site from the medical organizations, that is, medical personnel, hospital administrators, IT personnel, nurses, and telemedicine center managers. This small sample size is quite good in keeping with the nature of qualitative data. Findings by [33] reveal that results based on a small sample (under 10) tend to be unstable so for this reason a sample of 10 respondents was chosen. The researcher focused on individuals' interpretations of their environment and behavior (self and others), and the presentation of data lies in understanding the participants and their terms [34].The main purpose of qualitative research is to study a social reality [34]. In this case study, the focus will be on telemedicine projects selected from five SSA countries. The study focuses on how the firm works in relation to key success factors for sustainable outcomes of e-medicine.

### 3.4. Empirical Models and Method of Data Analysis

#### 3.4.1. Structural Equation Model

**Figure 1:**
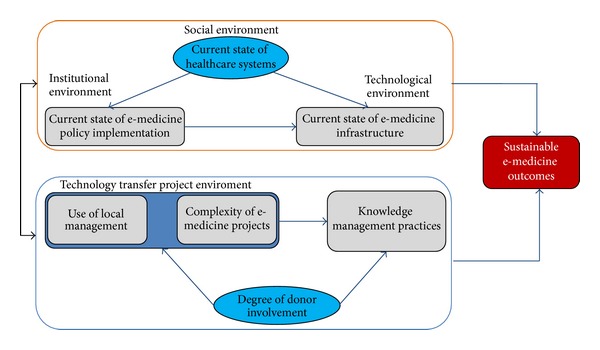
Hypothetical structural model for e-medicine sustainability.

In addition to qualitative analysis, in this study through a survey, respondents were asked to evaluate different statements on all the postulated domains in Figure 1. The respondents were asked to indicate their degree of agreement with the statements, using a seven-point Likert scale. Important to note is that the seven domains in Figure 1 (i.e., social environment; institutional environment; technological environment; degree of donor involvement; technological transfer environment; knowledge management environment; and e-medicine outcomes or e-medicine sustainability) are latent variables. Based on the responses, each item or statement for each domain can be analyzed separately or summed to create a score for a group of items or a summative scale. However, analyzing single-item responses pertaining to a latent variable (e.g., sustainability of e-medicine) is not reliable.

Generally, it is not advisable to make inferences based upon the analysis of single-item responses that are used in measuring a scaled latent variable. For this study, the format of the statements or responses can be either treated as ordinal or interval-level measurements. Responses to a single Likert item are normally treated as ordinal data because not all responses are equidistant. Such ordinal data can show how high the scores are from each other but may not indicate how much higher they are. When responses to several items are summed to measure a latent variable such as sustainability of e-medicine, all statements in a survey instrument use the same Likert scale; the responses are treated as interval data. This means that the interval between two points and the differences between each response are equal in distance.

The relationship of responses to statements or items and latent variables can be estimated using different procedures. Structural equation modeling was however adopted for this study. Structural equation model (SEM) allows both confirmatory and exploratory modeling, which is suited to both theory testing and theory development. Confirmatory modeling usually starts out with a hypothesis that gets represented in a causal model. The concepts used in the model must then be operationalized to allow testing of the relationships between the concepts in the model. The model is tested against the obtained measurement data to determine how well the model fits the data. The causal assumptions embedded in the model often have falsifiable implications which can be tested against the data [35]. With an initial theory, SEM can be used inductively by specifying a corresponding model and using data to estimate the values of free parameters. Often the initial hypothesis requires adjustment in light of model evidence.

The SEM was adopted due to the ability to construct variables, which are not measured directly but are estimated in the model from several measured variables, each of which is predicted to “tap into” the latent variables [36]. This allows the modeler to explicitly capture the unreliability of measurement in the model, which in theory allows the structural relations between latent variables to be accurately estimated.

In general, SEM is a combination of factor analysis and multiple regressions. The variables in SEM are measured (observed or manifested) variables (indicators or items in the survey instrument) and factors (latent variables) represented as seven domains in Figure 1. Variables and factors in SEM may be classified into endogenous/dependent variables or independent exogenous variables.

#### 3.4.2. Model Specifications

SEM is used as a confirmatory technique; researchers [35] suggest that when building the correct model, the researcher uses two different kinds of variables, namely, exogenous and endogenous variables. The distinction between these two types of variables is whether the variable regresses on another variable or not [37]. In SEM, other variables regress on exogenous variables. Exogenous variables can be recognized in a graphical version of the model, as the variables sending out arrowheads, denoting which variable it is predicting. A variable that regresses on a variable is always an endogenous variable, even if this same variable is also used as a variable to be regressed on. Endogenous variables are recognized as the receivers of an arrowhead in the model. In this study, we have institutional environment (IE), technical environment (TE), technology transfer project environment (TTE), knowledge management practices (KM), social environment (SE), and donor involvement (DI) as exogenous variables or independent variables, while sustainable e-outcome (ST) is the dependent variable (DV).

The measured variables are within rectangles and the names of factors or latent variables are ellipses. Rectangles and ellipses are connected with lines having an arrowhead on one end (unidirectional causation) or two (implying no specification of direction on causality). Dependent variables are those which have one-way arrows pointing to them and independent variables are those which do not. Dependent variables have residuals (denoted as e). The residual is not perfectly related to the other variables in the model and is indicated by arrow pointing to measured or latent variables. Therefore, a line with an arrow at both ends indicates a covariance between the two variables, with no implied direction of effect. An arrow pointing to each measured variable implies that the factor does not predict the measured variable perfectly. There is variance (residual) in the measured variable that is not accounted for by the factor. The structural model is illustrated below.

#### 3.4.3. Interpretation of Model and Discussion

All model constructs were estimated by R package Lavaan. The R package Lavaan is a free, open-source R package for latent variable analysis. You can use Lavaan to estimate a large variety of multivariate statistical models, including path analysis, confirmatory factor analysis, structural equation modeling, and growth curve models. According to the author [38], the Lavaan package was developed to provide users with a free, open-source but commercial-quality package for latent variable modeling. The long-term goal of Lavaan is to implement all the state-of-the-art capabilities that are currently available in commercial packages.

Two confirmatory data analysis models were estimated for each country. As explained before, the first model was for testing institutional, technical, and social environmental variables relationships. The second model was testing donor involvement, technology transfer project environment, and knowledge management practices relationship. Each model was estimated as a stack of system of equations and used the “GROUP” option in Lavaan to get the estimate of the model at the country level.

We used four different measures to test the model fit. The comparative fit index (CFI) assesses fit relative to other models and uses an approach based on the noncentral parameters distribution with noncentrality parameter distribution. The larger the value CFI is, the better the model fit is. The CFI values greater than .9 often are indicative of good fitting models. The RMSEA estimates the lack of fit in a model compared to a perfect or saturated model. Essentially, RMSEA is a measure of noncentrality relative to sample size and degrees of freedom. For a given noncentrality, large numbers of observations and degrees of freedom imply a better fitting model, that is, a smaller RMSEA. Values of .06 or less indicate a close-fitting model. Values larger than .10 are indicative of poor-fitting models. However, the index is less preferable with small samples. Other test statistics used in the study were Chi-square and the likelihood ratio test.

## 4. Results and Discussion

### 4.1. Confirmatory Analysis for the Model

The fit test statistics for the model are as follows: CFI (0.91), RMSEA (0.1) with a 90% confidence interval of 0.086 and 0.113, the standardized root mean square residual was 0.076, and the Chi-square and log of likelihood that test the null hypothesis that all parameters are equal to zero was significant to the 1% level. The hypothesized model appears to be a good fit to the data. There was no need to conduct post hoc modifications of the model because of the good fit of the data to the model.

Tables 2–4 show the estimated coefficients for Ethiopia, Uganda, and Nigeria confirmatory factor analysis as related to institutional environment, technological environment, and social environment. In the tables, estimates are estimated parameters, Std.err is standard error, and std.lv and Std., respectively, represent estimates of the model when items are standardized and the latent variables are not standardized and when both variables are standardized. The latter is often called the completely standardized solution.

In the first column of Tables 2–4, parameters of items are unstandardized, and the first item in each domain is fixed to 1 to allow model identification. The associated, therefore, values and probabilities are not estimated. The estimated values are equivalent to factor loading for each item. If the unstandardized parameter estimates are divided by their respective standard errors, a *Z* score is obtained for each estimated parameter. The *Z* score is used because the standard errors are adjusted for nonnormality. The probability values generated using the *Z*-score are used to test the null hypothesis that the unstandardized regression coefficients are equal to zero.

Apart from factor loadings, covariances between latent variables and variances (residual variances) are also reported. Loadings show the effect of latent variable on the measure; if a measure loads on only one factor, the standardized loading is the measure's correlation with the latent variables and can be interpreted at the square root of the measure's reliability. In the tables, the variance (or the residual variance) in the measure is not explained by the latent variable; error variance does not imply that the variance is random or not meaningful, just that it is unexplained by the latent variables. To estimate the standardized models, the factor variance is set equal to one and all the loadings are free to vary. For unstandardized models, as mentioned before, one of the loadings is set to one (called the marker variable), the others are free, and the factor variance is free. Covariances between latent variables measure how much two latent variables change together. However, standardized covariances are easy to understand, as they are based on the same distribution and scale.

Results of the model for Ethiopia (Table 2) indicate that all item loadings were statistically significant (*P* value < 0.01). Therefore, we reject the null hypothesis and accept the alternative that all items included in the model influence the respective latent variables. Likewise, the residual variances are statistically significant at (*P* value < 0.01), rejecting the null hypothesis that the items are measured without errors. It can also be seen that there is a statistically significant positive covariation (*P* value < 0.05) between institutional environment and technological environment. This means that the two latent variables influence each other. There was a positive covariation between Institutional Environment involvement and Social Environment and Technological environment; however, it was not statistically significant. The results showing a positive covariance between technological environment and social environment were not statistically significant.

Based on these results, the standardized loadings (last column) indicate the importance of each item at loading on the respective latent variables. For example, IE1 with a loading factor of 0.947 was more important than IE3 with a loading factor of 0.922. The item IE5 was the least important, with a loading factor of 0.361.

During our interviews with key stakeholders, technological environment was echoed as an important factor in institutional environment for e-medicine sustainability. A doctor in Uganda argued, “We use computers for different reasons citing the pharmacy and laboratories, the doctor said they don't have a strategy; everything is done on a piece-meal basis, with IT technicians only helping in trouble shooting.”

The model results for Uganda (Table 3) also reveal that all item loadings were statistically significant (*P* value < 0.01). Therefore, we reject the null hypothesis and accept the alternative that all items included in the model influence the respective latent variables. The results also show that, except for TE3, all residual variances were statistically significant at *P* value < 0.01. The null hypothesis that the items are measured without errors is therefore rejected. It can also be seen that there was a statistically significant positive covariation (*P* value < 0.05) between institutional environment and technological environment. This means that the two latent variables influence each other. There was positive nonstatistical significant covariation between institutional environment and social environment.

Based on these results, the standardized loadings (last column) indicate the importance of each item at loading on the respective latent variables. For example, Uganda SE2 with a loading factor of 0.714 was more important than SE3 with loading factor of 0.578. The item SE1 was the least important, with a loading factor of 0.407.

The model results for Nigeria (Table 4) also reveal that all item loadings were statistically significant (*P*-value < 0.01). Therefore, we reject the null hypothesis and accept the alternative that all items included in the model influence the respective latent variables. The results also indicate that most of the residual variances were statistically significance at *P*-value < 0.01 apart from SE 2 (*P*-value < 0.01 = 0.064). We therefore reject the null hypothesis that the items are measured without errors. It can also be seen that there was a statistically significant positive covariation (*P*-value < 0.05) between institutional environment, technological environment, and social environment. This means that the two latent variables influence each other. There was a positive institutional environment and social environment relationship between and statistically significant.

Based on these results, the standardized loadings (last column) indicate the importance of each item at loading on the respective latent variables. For example, in Nigeria, SE2 with a loading factor of 0.967 was more important than SE1 with a loading factor of 0.895. The item SE3 was the least important with a loading factor of 0.450.

### 4.2. Discussion and Framework Development

As mentioned earlier in the study, applying e-medicine concepts in SAA has been a pressing issue [8, 39]. This study sought to develop a framework which would facilitate the development, implementation, and sustainability of e-medicine in Sub-Sahara Africa. The results presented in the study not only show how technological, institutional social environmental, technology transfer environment, and donor involvement factors have impacted on sustainable e-medicine transfer but also the impacts of knowledge management on sustainable e-medicine transfer outcomes.

#### 4.2.1. Social Environment

The revamping of contemporary healthcare practices and system from their present glut of bureaucracy, corruption, and social stigmas to a coordinated flow of materials and information has been a major problem in SSA. However, the potential effectiveness of telemedicine can no longer be taken for granted. This is, as the results of the study indicate, a healthy technology transfer domain and telemedicine projects supported by a more robust national healthcare system are likely to produce more favorable outcomes compared to telemedicine projects that lack support of a robust healthcare system.

The results of this study showed that the social environment strongly influenced the level of institutional and technological environments on sustainable e-medicine outcomes.

The social environment as far as technology transfer is concerned has been supported by many scholars. For instance, [8] argue that although general ICT policies have little effect on the success of e-medicine in SSA, policies that are specifically targeted at e-medicine were necessary if telemedicine has to provide meaningful results. Also, while looking at technology adoption at the individual level of analysis, [40] tested a comprehensive model of the moderating effects of national cultural dimensions on technology transfer and found that all dimensions of culture [41] had effects on decisions to use technology. In their studies, Straub and others found that both cultural subconstructs have a mediating effect on IT implementation [42, 43]. In other words, beliefs, values, and culturation affect the effectiveness of IT implementation, in addition to their direct effects on IT outcomes.

**Figure 2:**
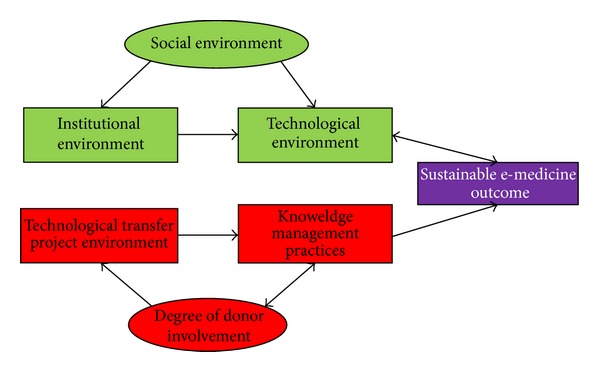
Framework for e-medicine sustainability.

The purpose of the study was to develop a framework which will facilitate the development, implementation, and sustainability of e-medicine in Sub-Saharan Africa; the study therefore presents the tested model as per results of the study as illustrated in Figure 2.

## 5. Conclusion

### 5.1. Policy Contributions and Implications of the Study

This study contributes to the methodological discourse in information science and social sciences and on action research. We identify vital, yet underdeveloped, quality criteria for action research in information science and social science model for the sustainability of interventions. The researcher was against the notion of action research projects that end up with changes that last only as long as the attention of action researchers remains or, similarly, end up with a prototype but never routinely used systems. The study postulates a model for sustainability of e-medicine that is much needed in sub-Saharan Africa.

Our findings suggest a roadmap for being deliberate about sustainability efforts by virtue of the development and implementation of a sustainability plan in e-medicine. Intention is particularly important in light of research that discusses how early sustainability planning is an important step toward actually sustaining programs and that calls for the need to be ethically responsible for continuing programs once begun, particularly for those in the neediest communities.

The study recommends program teams to develop, implement, and monitor a sustainability plan which was not found consistent in the organizations where the study was done. Training should be a major component for sustainability, and continuous assessment of e-medicine programs in the country sites should be embraced both at the individual level and at the organizational level. Sustaining initiatives is a process that benefits from continual monitoring and adaptation to meet individual, family, program, and community needs. The sustainability conceptual model developed will help in providing e-medicine professionals with grounded, reliable, and valid information on which to build their sustainability efforts in an intentional, cohesive, comprehensive, and efficient way.

The central contribution of this work is a call to IS researchers and telemedicine practitioners to extend the sustainability of information systems to the developing world as a part of our communal research agenda. The study highlighted this need specifically by outlining a research framework for telemedicine sustainability in Sub-Saharan Africa; the perspectives offered are by no means limited to the specific region but are applicable for the developing world with a fragile healthcare system. This research will set a stage for IS researchers to continue in this vein by developing and augmenting IS theories to examine the sustainability interplay of ICTs in societies. The study proposes an inductive ICT transfer framework that researchers can institute to investigate myriad factors that lead to and influence ICT transfers in the developing world. The development of this theoretical framework is a step towards creating a new reference frame of social development: one that is parallel to the mature phenomenon of organizational development.

## Figures and Tables

**Table 1:** Comparison of traditional and constructivist criteria.

Traditional criteria	Trustworthiness criteria	Measurement of criteria
Internal validity	Credibility	(i) Extended engagement in the field (ii) Variety of data types (iii) Peer debriefing

External validity	Transferability	(i) Purposive and theoretical sampling (ii) Detailed description

Objectivity	Conformability	Meticulous data management and recording

Reliability	Dependability	(i) Snow balling (ii) Accurate records maintained (iii) Participants confidentiality protected

**Table 2:** Confirmatory factor analysis results for Ethiopia on institutional, technical, and social environmental variables relationship.

Variable	Estimate	Std.err	*Z* value	*P* (>|*z*|)	Std.lv	Std.all
Institutional environment						
IE1	1.000				1.456	0.946
IE2	0.946	0.046	20.548	0.000	1.377	0.951
IE3	0.696	0.074	9.348	0.000	1.013	0.661
IE4	0.706	0.067	10.591	0.000	1.028	0.712
IE5	0.567	0.078	7.254	0.000	0.826	0.556
Technological environment						
TE1	1.000				0.613	0.473
TE2	1.658	0.304	5.45	0.000	1.016	0.758
TE3	2.224	0.435	5.116	0.000	1.362	0.978
Social environment						
SE1	1.000				0.998	0.81
SE2	0.985	0.096	10.31	0.000	0.983	0.872
SE3	0.792	0.102	7.771	0.000	0.791	0.664
SE4	0.823	0.096	8.546	0.000	0.821	0.72
Covariance among latent variables					
Institutional environment to						
technical environment	0.33	0.107	3.081	0.002	0.37	0.37
social environment	0.57	0.153	3.729	0.000	0.392	0.392
Technical environment to						
social environment	0.206	0.074	2.793	0.005	0.336	0.336
Variances						
IE1	0.248	0.066	3.746	0.000	0.248	0.105
IE2	0.199	0.058	3.434	0.001	0.199	0.095
IE3	1.32	0.17	7.783	0.000	1.32	0.563
IE4	1.025	0.133	7.68	0.000	1.025	0.493
IE5	1.522	0.192	7.916	0.000	1.522	0.691
TE1	1.305	0.167	7.815	0.000	1.305	0.777
TE2	0.766	0.146	5.233	0.000	0.766	0.426
TE3	0.086	0.2	0.43	0.668	0.086	0.044
SE1	0.524	0.092	5.698	0.000	2.524	0.345
SE2	0.304	0.071	4.268	0.000	0.304	0.239
SE3	0.791	0.11	7.166	0.000	0.791	0.558
SE4	0.627	0.092	6.811	0.000	0.627	0.482
IE	2.119	0.297	7.141	0.000	1	1
TE	0.375	0.138	2.723	0.006	1	1
SE	0.996	0.188	5.3	0.000	1	1

**Table 3:** Confirmatory factor analysis for Uganda on the institutional, technical, and social environmental variables relationship.

Variable	Estimate	Std.err	*Z* value	*P* (>|*z*|)	Std.lv	Std.all
Institutional environment						
IE1	1.000				1.488	0.947
IE2	0.506	0.071	7.135	0.000	0.753	0.556
IE3	0.987	0.066	14.910	0.000	1.468	0.922
IE4	0.383	0.085	4.532	0.000	0.570	0.381
IE5	0.390	0.091	4.273	0.000	0.581	0.361
Technological environment						
TE1	1.000				0.553	0.415
TE2	1.540	0.496	3.104	0.002	0.851	0.679
TE3	0.868	0.309	2.807	0.005	0.480	0.414
Social environment						
SE1	1.000				0.669	0.407
SE2	1.284	0.362	3.546	0.000	0.859	0.714
SE3	1.101	0.319	3.448	0.001	0.736	0.578
SE4	1.098	0.338	3.248	0.001	0.734	0.495
Covariance among latent variables						
Institutional environment to						
technical environment	0.388	0.143	2.706	0.007	0.472	0.472
social environment	0.299	0.132	2.271	0.023	0.300	0.300
Technical environment to						
social environment	0.189	0.085	2.222	0.026	0.511	0.511
Variances						
IE1	0.248	0.066	3.746	0.000	0.248	0.105
IE2	0.199	0.058	3.434	0.001	0.199	0.095
IE3	1.320	0.170	7.783	0.000	1.320	0.563
IE4	1.025	0.133	7.680	0.000	1.025	0.493
IE5	1.522	0.192	7.916	0.000	1.522	0.691
TE1	1.305	0.167	7.815	0.000	1.305	0.777
TE2	0.766	0.146	5.233	0.000	0.766	0.426
TE3	0.086	0.200	0.430	0.667	0.086	0.044
SE1	0.524	0.092	5.698	0.000	0.524	0.345
SE2	0.304	0.071	4.268	0.000	0.304	0.239
SE3	0.791	0.110	7.166	0.000	0.791	0.558
SE4	0.627	0.092	6.811	0.000	0.627	0.482
IE	2.119	0.297	7.141	0.000	1.000	1.000
TE	0.375	0.138	2.723	0.006	1.000	1.000
SE	0.996	0.188	5.300	0.000	1.000	1.000

**Table 4:** Confirmatory factor analysis results for Nigeria on the institutional, technical, and social environmental variables relationship.

Variable	Estimate	Std.err	*Z* value	*P* (>|*z*|)	Std.lv	Std.all
Institutional environment						
IE1	1.000				1.399	0.907
IE2	1.029	0.063	16.435	0.000	1.440	0.955
IE3	0.452	0.086	5.275	0.000	0.632	0.414
IE4	0.608	0.067	9.114	0.000	0.851	0.639
IE5	0.476	0.077	6.170	0.000	0.666	0.473
Technical environment						
TE1	1.000				0.775	0.704
TE2	1.125	0.114	9.834	0.000	0.873	0.866
TE3	1.259	0.127	9.920	0.000	0.976	0.930
Social environment						
SE1	1.000				1.171	0.895
SE2	1.020	0.059	17.335	0.000	1.195	0.967
SE3	0.575	0.099	5.827	0.000	0.674	0.450
SE4	0.701	0.059	11.953	0.000	0.821	0.759
Covariance among latent variables						
Institutional environment to						
technological environment	0.278	0.102	2.735	0.006	0.256	0.256
social environment	0.281	0.144	1.950	0.051	0.172	0.172
Technical environment to						
social environment	0.265	0.086	3.074	0.002	0.292	0.292
Variances						
IE1	0.422	0.096	4.411	0.000	0.422	0.177
IE2	0.199	0.090	2.223	0.026	0.199	0.088
IE3	1.930	0.226	8.547	0.000	1.930	0.828
IE4	1.052	0.127	8.258	0.000	1.052	0.592
IE5	1.538	0.181	8.501	0.000	1.538	0.776
TE1	0.610	0.079	7.727	0.000	0.610	0.504
TE2	0.254	0.052	4.850	0.000	0.254	0.250
TE3	0.149	0.057	2.630	0.009	0.149	0.135
SE1	0.342	0.064	5.366	0.000	0.342	0.200
SE2	0.098	0.053	1.853	0.064	0.098	0.064
SE3	1.790	0.210	8.545	0.000	1.790	0.798
SE4	0.495	0.063	7.879	0.000	0.495	0.424
IE	1.957	0.283	6.924	0.000	1.000	1.000
TE	0.601	0.126	4.771	0.000	1.000	1.000
SE	1.372	0.200	6.849	0.000	1.000	1.000
